# Thermoacidophilic *Alicyclobacillus* Superoxide Dismutase: Good Candidate as Additives in Food and Medicine

**DOI:** 10.3389/fmicb.2021.577001

**Published:** 2021-03-18

**Authors:** Xueqian Dong, Wei Wang, Shannan Li, Hongyu Han, Peiwen Lv, Chunyu Yang

**Affiliations:** ^1^State Key Laboratory of Microbial Technology, Institute of Microbial Technology, Shandong University, Qingdao, China; ^2^Shandong Food Ferment Industry Research & Design Institute, QiLu University of Technology (Shandong Academy of Sciences), Jinan, China

**Keywords:** thermoacidophilic *Alicyclobacillus* strain, superoxide dismutase, acid tolerant, thermostability, cambialistic Fe/Mn type

## Abstract

Thermoacidophilic *Alicyclobacillus* strains attract great interests as the resource of thermostable or acidic enzymes. In this study, a putative gene encoding superoxide dismutase (*Aa*SOD) was identified in a thermoacidophilic *Alicyclobacillus* strain. With a 16-fold activity observed, the *Aa*SOD activity expressing in the medium of manganese enrichment was much higher than that in the iron medium. In addition, the purified *Aa*SOD can be reconstituted exclusively with either Fe^2+^ or Mn^2+^, with its Mn-bound protein showing 25-fold activity than that of Fe-bound form. The optimal temperature for *Aa*SOD reaction was 35°C, and was highly stable at any certain temperature up to 80°C. Of particular interest, the enzyme is found to be very stable across a wide pH range spanning from 2.0 to 10.0, which confers its robust stability in the acidic stomach environment and implies striking potentials as food additive and for medical use.

## Introduction

Superoxide dismutases (SODs, EC 1.15.1.1) are one type of antioxidant enzymes derived from living organisms which can protect themselves against oxidative stress, by achieving disproportionation of superoxide anion radical (O_2_^–^) to hydrogen peroxide (H_2_O_2_) and dioxygen (O_2_) through a redox cycle of metal ions (Abreu and Cabelli, 2010). SODs have been confirmed to be closely related to various physiological processes in living bodies, since they are related not only to the prevention and treatment of various diseases, but also to anti-aging therapy and prevention of skin pigmentation. SODs, also known as metalloenzymes, are divided into four groups depending on their metal preferences: manganese SOD (Mn-SOD), iron SOD (Fe-SOD), copper/zinc SOD (Cu/Zn-SOD), and nickel SOD (Ni-SOD) (Perry et al., 2010; Bafana et al., 2011). The resolved crystal structures of different types of SODs revealed that these proteins differ not only with regard to the coordinated metal ion, but also to the protein folding. Mn-SOD and Fe-SOD are closely related when compared by their amino acids and tertiary structures, and assigned into one family (Miller, 2012). In this family, some use both Fe^2+^ and Mn^2+^ as their cofactors, and thus are referred to as the cambialistic Fe/Mn type SODs (Schmidt et al., 1996; Yamano et al., 1999). In spite of metal ion specificity, these Fe-SODs, Mn-SODs, and the cambialistic SODs were found to be phylogenetically related, which is proved by the high sequence identity, as well as many common features in their tertiary structures (Jackson and Cooper, 1998). In strain *Escherichia coli*, the Mn-SOD and Fe-SOD are extremely similar in sequence (45% identity) and crystal structure (91% homology), but are strictly specific to their cognate metal ions (Hunter et al., 2018). The cambialistic SODs can accommodate both Fe^2+^ and Mn^2+^ as their cofactors for catalysis but display different ions preference. ApeSOD from *Aeropyrum pernix* is less active in its Fe-bound form while the cambialistic SOD from *Propionibacterium shermanii* exhibits similar enzymatic activity in the presence of Fe^2+^ and Mn^2+^ (Meier et al., 1982; Nakamura et al., 2011). Comparison of the crystal structures of both SODs shows that these two cambialistic SODs have different binding forms with Fe^2+^ and Mn^2+^, which was proposed as a structural explanation for their ions preference (Schmidt et al., 1996; Nakamura et al., 2011).

Supplementation of exogenous SOD has been reported to boost the antioxidant defense of host (Manolov et al., 2017). Considerable evidences from clinical or animal models have been accumulated and revealed that SOD is beneficial in a wide variety of applications, including reduction fibrosis following radiation treatment, preventions of aging, diabetes, tumor formation, and hepatitis C related fibrosis (Matès and Sánchez-Jimènez, 2000; Emerit et al., 2006; Bafana et al., 2011; Naso et al., 2011), or reduction of the cytotoxic and cardiotoxic effects of anticancer drugs (Trotti, 1997). Due to its excellent antioxidant and therapeutic properties, various SOD products have been applied in the industries of medicine, health care products, food additives, and cosmetics (Johnson and Giulivi, 2005). However, the thermal denaturation is an important factor leading to enzyme inactivation because the high temperature treatment is often processed in practical applications of the enzyme (Song et al., 2009). Therefore, it has stimulated a widespread interest in exploring thermotolerant enzymes from thermophilies (Morozkina et al., 2010). A plenty of previous research confirmed that the thermostable enzymes have unique structural and functional characteristics that give them good adaptability in high temperature environment. To date, SODs have been identified from various thermophilic archaea and bacteria, including *Bacillus* (Boyadzhieva et al., 2010), thermoacidophilic crenarchaeon (Slutskaya et al., 2012), *Pyrobaculum* (Amo et al., 2003), *Chloroflexus* (Lancaster et al., 2004), *Thermus* species (Mandelli et al., 2013) and so on. In addition, SODs from thermophilic fungi have also been reported by a few studies, such as *Thermomyces lanuginosus* Mn-SOD (Li et al., 2005) and *Thermoascus aurantiacus* var. *levisporus* Cu/Zn-SOD, which also display remarkable tolerance to the extreme temperature (Song et al., 2009). Besides these wild type thermophilic SODs, thermostable SODs were also constructed by engineering protocols. The heat-resistance and stress-tolerance of thermophilic cambialistic Fe/Mn-SOD from *A. pernix* K1 was successfully developed through the fusion with the N-terminal domain of *Geobacillus thermodenitrificans* SOD (Li et al., 2017).

Comparing with its thermostability, the acidic tolerance of SOD is far less addressed, since few species of SOD are enzymatically stable under extremely acidic conditions (Ken et al., 2005). *Alicyclobacillus* strains are heterotrophic organisms that mostly inhabit acidic geothermal environments such as hot springs and acid mine waters (Zhang et al., 2015; López et al., 2018). As an extraordinary resource for exploring unique enzymes with acidophilic and (or) thermophilic properties, the *Alicyclobacillus* isolates are of great interests in recent years because of their double physiological characteristics of acidophilic and thermotolerant (McKnight et al., 2010). Some thermostable and acidic tolerant glycoside hydrolases, including α-amylase (Zhang et al., 2018), glucanase (Bai et al., 2010), xylanase (Lee et al., 2018) etc., have been identified from *Alicyclobacillus* species. Correa-Llantén et al. (2014) have purified SOD from *Alicyclobacillus* isolate CC2 that was most active at 55°C, pH 7.4. However, there are no further studies performed on *Alicyclobacillus*-derived SOD. In the present study, we engaged in exploring a novel SOD from a strain *Alicyclobacillus* sp. HJ previously isolated from the hot-spring (Zhang et al., 2018). As a result, the Fe/Mn-SOD with remarkable acid and thermal tolerance was identified and catalytically characterized in this study.

## Materials and Methods

### Strains and Plasmids

Strain *Alicyclobacillus* sp. HJ was isolated from Tengchong hot-spring, Yunnan, China and deposited in the Marine Culture Collection of China (MCCC 1K03506). The 16S rDNA sequence analysis revealed 100% similarity with gene of *Alicyclobacillus acidocaldarius* DSM451. In the M63 medium, the strain grows optimally at conditions of 65°C and pH 4.0 (Zhang et al., 2018). The clone vector pEASY-blunt and *E. coli* DH5α was used for gene cloning and fidelity confirmation. The recombinant expression vector pET-24a(+) with *Aa*SOD encoding gene was transformed into *E. coli* BL21 CodonPlus for protein expression, and Luria-Bertani (LB) medium was used for the cultivation of recombinants.

### Phylogenetic Analysis of *Aa*SOD and Modeling

The amino acid sequence of *Aa*SOD were submitted to National Center for Biotechnology Information (NCBI) for blast analysis. A phylogenetic tree was constructed to establish the evolutionary relationship among *Aa*SOD and corresponding SOD sequences retrieved from the NCBI protein database. MEGA 7.0 software was used to build the phylogenetic tree, by using Neighbor-Joining method with bootstrap replications of 1,000 (Kumar et al., 2016). Amino acid sequences multiple alignment was performed by ClustalX 2.0 and DNAMAN programs. The protein sequence of *Aa*SOD was submitted to SWISS-MODEL server for homology modeling (Waterhouse et al., 2018).

### Protein Expression and Purification

Genomic DNA of *Alicyclobacillus* sp. HJ was isolated with ChargeSwitch^®^ gDNA Mini Bacteria Kit (Life Technologies) and used as template for *Aa*SOD gene amplification. Primers for its PCR were designed as *Aa*SOD-F (5′ –CGGGATCCATGCCA CATCAACTCCCAC–3′) and *Aa*SOD-R (5′ –CCCAAGCTTGC CGTTCAGCGCGGCCTCGT–3′), which incorporate restriction sites *Bam*HI and *Hin*dIII (underlined), respectively. The PCR products was ligated into pET-24a(+) and transformed in the competent cells *E. coli* BL21 CodonPlus. The correct clones were picked and further verified by PCR sequencing.

For *Aa*SOD expression, cells of *E. coli* harboring pET-24a(+)-*Aa*SOD were cultured in 5 ml LB medium at 37°C for 12 h. Subsequently, the culture was diluted at the ratio of 1:100 into 100 ml fresh LB media containing 50 mg l^–1^ ampicillin and grown at 37°C, 180 rpm. When OD_600_ of the culture reached to 0.6, isopropyl-β-D-thiogalactoside (IPTG) was added up to the final concentration of 0.5 mM for protein induction. After cultivation at 18°C, 100 rpm for 12 h, cells were harvested by centrifugation at 8,000 × *g* for 10 min at 4°C. The cell debris was resuspended in 10 ml buffer A (20 mM Tris–HCl, 0.5 M sodium chloride, pH 8.0) with a protease inhibitor cocktail and disrupted using a high-pressure homogenizer (ATS Engineering Inc., Canada). The cell lysis was then centrifuged at 17,000 × *g* for 30 min at 4°C, and the supernatant was applied to HisTrap^TM^ FF crude column (GE Healthcare) for protein purification at 4°C. The target protein was eluted with buffer A that contains 250 mM imidazole. For further analysis, the elution was concentrated and dialyzed overnight with 50 mM Tris–HCl buffer (pH 8.0) at 4°C. The protein concentration was determined by Brad-Ford method with bovine serum albumin (BSA) as standard (Bradford, 1976). The expression level and purity of target protein were verified by SDS-PAGE (12% w/v), with Bio-Rad Mini-PROTEAN TETRA electrophores system (United States).

### SOD Activity Assay

The superoxide dismutase activity was measured by pyrogallic acid spontaneous oxidation assay (Marklund and Marklund, 1974). Briefly, 50 mM pyrogallic acid was added to 50 mM Tris–HCl buffer (pH 8.0) at the final volume of 3 ml reaction mixture. The absorbance of the mixture was then measured at 325 nm at every 30 s, during a 4-min reaction process at room temperature. The rate of the self-oxidation was approximately kept at 0.07 OD min^–1^ and a certain amount *Aa*SOD was added to the reaction mixture at an inhibition ratio of 50%. One unit of superoxide dismutase activity was defined by the amount of enzyme that inhibited the rate of pyrogallic acid spontaneous oxidation by 50%. The activities of each sample were performed in three replicates.

### Influence of Fe^2+^ and Mn^2+^ on the Native Protein Expression

The *E. coli* strain carrying pET-24a(+)-*Aa*SOD was prepared as above described and then inoculated into 100 ml LB medium, or LB media containing 1 mM FeCl_2_, MnCl_2_, or both ions. After IPTG induction for 12 h, cells were collected by centrifugation and lysed in high-pressure homogenizer. After centrifugation, the supernatant flowed through the HisTrap crude column for protein purification and then the activity of SOD was measured by standard procedures described above.

### Reconstitution of Metals Into SOD

The apo-enzyme was prepared as previous described (He et al., 2007). In brief, the enzyme was dialyzed with 50 mM acetate buffer (pH 3.8) containing 8 M urea and 10 mM EDTA for 16 h at room temperature. Then the enzyme was dialyzed at 4°C sequentially with 50 mM acetate buffer (pH 3.8) containing 8 M urea, 50 mM Tris–HCl buffer (pH 7.0) containing 8 M urea, and 50 mM Tris–HCl buffer (pH 8.0). To prepare Mn- or Fe- reconstituted *Aa*SOD, the apo-protein was subsequently dialyzed for 4 h at 4°C with the following buffers: 8 M urea, 50 mM acetate buffer, 10 mM MnSO_4_ and (or) FeSO_4_, pH 3.8; 8 M urea, 50 mM phosphate buffer, 10 mM MnSO_4_ and (or) FeSO_4_, pH 7.0; 4 M urea, 50 mM phosphate buffer, 10 mM MnSO_4_ and (or) FeSO_4_, pH 7.0; 2 M urea, 50 mM phosphate buffer, 10 mM MnSO_4_ and (or) FeSO_4_, pH 7.0; 50 mM phosphate buffer, 1 mM MnSO_4_ and (or) FeSO_4_, pH 7.0; and 50 mM Tris–HCl buffer/0.5 mM EDTA, pH 8.0, respectively. The reconstituted enzymes obtained were analyzed by SDS-PAGE and the specific activity was determined by pyrogallic acid spontaneous oxidation assay.

### Metal Analysis

Inductively Coupled Plasma Mass Spectrometry (ICP-MS) was used to measure the contents of Fe^2+^ and Mn^2+^ Briefly, the mixture of 500 μl sample (1 mg ml^–1^) and 8 ml 65% nitric acid was digested by microwave digestion system. ddH_2_O was added to the mixture at a total volume of 10 ml subsequently and further analyzed by ICP8000 ICP-OES. The Fe^2+^ and Mn^2+^ concentrations were calculated based on their corresponding standard curves. Under experimental conditions, the standard recovery rate is between 96.23–102.65% for Fe^2+^, and 98.31–103.05% for Mn^2+^.

### Effect of Temperature on *Aa*SOD

The optimum temperature was determined by measuring the activity of SOD at different temperatures ranging from 25 to 80°C. For thermal stability measurement, 1 mg ml^–1^ protein was incubated in 50 mM Tris–HCl buffer (pH 8.0) at room temperature (RT), 60, 70, 80, 90, and 100°C for 1 h, respectively. Subsequently, the residual activities were determined by the standard condition (pH 8.0, 25°C) as described above. Each temperature was performed in triplicate. The relative activity was calculated by the percentage of the maximum activity at 35°C.

After dialyzed overnight with 200 mM HEPES (pH 8.0), the purified *Aa*SOD was tested for its thermal denaturation in the MicroCal VP-DSC (Malven). Protein sample (1 mg ml^–1^) was stirred to degassed under vacuum, cooled down to 20°C, and then gradually heated to 110°C. Using the dialysis buffer as a baseline, *Aa*SOD sample was scanned from a temperature range of 70 to 110°C at a rate of 1.5°C min^–1^. By subtracting the baseline, the transition curve was fitted by non-2-state model and the *T*_*m*_ value was calculated (MN2state) in the Origin software (MicroCal Software, Inc., Northampton, MA, United States).

### pH Tolerance of *Aa*SOD

The pH stability of *Aa*SOD was determined by incubating the enzyme in various buffers with different pH values at 25°C for 1 h, and then measuring the residual activity under standard assay conditions described above. The buffer systems include 200 mM KCl-HCl buffer (pH 1.0–2.0), acetate buffer (pH 2.0–5.0), sodium phosphate buffer (pH 5.0–7.0), Tris–HCl buffer (pH 7.0–9.0), and glycine–NaOH buffer (pH 9.0–10.0). Each experiment was performed in triplicate. The maximal activity of *Aa*SOD was defined as 100% for relative activity calculation.

### Effects of Divalent Metal Ions on SOD Activity

Effects of metal ions on *Aa*SOD activity were determined by adding various divalent metal salts (MgCl_2_, ZnSO_4_, BaCl_2_, CaCl_2_, CuSO_4_, NiSO_4_, CoCl_2_, MnSO_4_, and FeSO_4_), at a final concentration of 1 mM in 50 mM Tris–HCl buffer (pH 8.0). After incubating the purified *Aa*SOD with each ion at 25°C for 30 min, the activities were measured under the standard conditions. The activity of the enzyme without addition of metal ions was defined as 100% and used for relative activity calculation.

### Statistical Method

The data was analyzed using origin 9.0 (OriginLab corp., United States), the means and standard deviations were calculated by descriptive statistics.

### Nucleotide Sequence Accession Number

The nucleotide sequence encoding *Aa*SOD was deposited in the GenBank database under accession number of MT559075.

## Results and Discussion

### Sequence Analysis and Structural Analyses of *Aa*SOD

**FIGURE 1:**
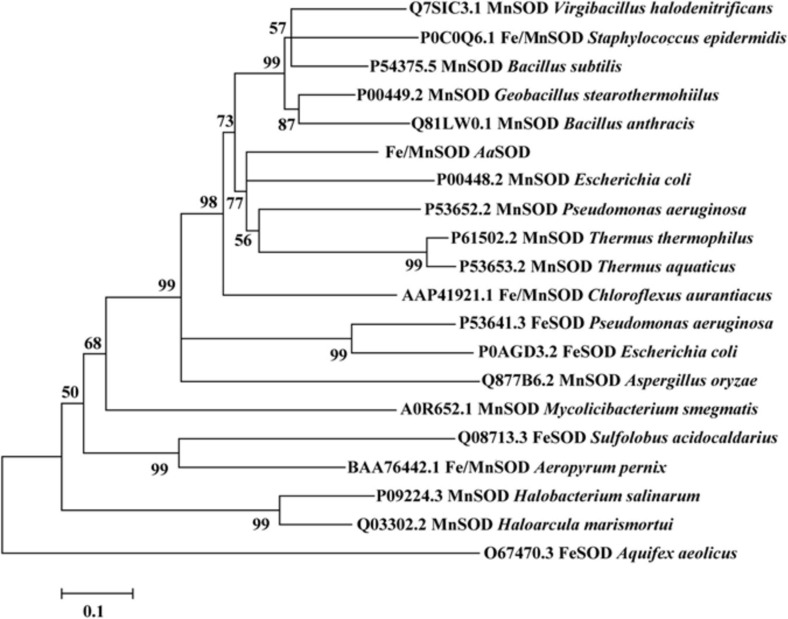
Neighbor-joining phylogenetic tree based on the amino sequences of *Aa*SOD and its closest relatives retrieved from NCBI database. The tree was built by the program MEGA version 7.0. Bootstrap percentages >50% (based on 1000 replications) are shown at branch points.

**FIGURE 2:**
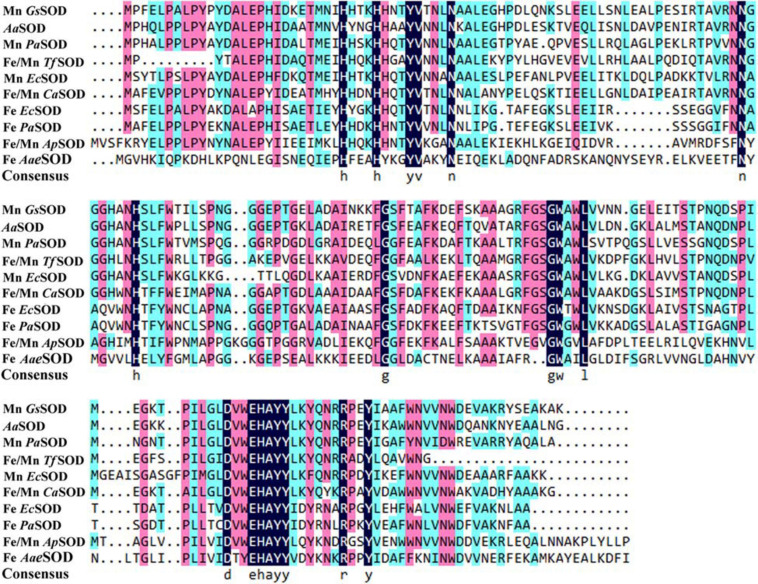
Multiple alignment of *Aa*SOD with other identified Fe, Mn, or Fe/Mn type SODs that retrieved from NCBI database. *Gs*SOD: *Geobacillus stearothermophilus*, *Aa*SOD: this study, *Pa*SOD: *Pseudomonas aeruginosa*, *Tf*SOD: *Thermus filiformis*, *Ec*SOD: *Escherichia coli*, *Ca*SOD: *Chloroflexus aurantiacus*, *Ap*SOD: *Aeropyrum pernix*, *Aae*SOD: *Aquifex aeolicus*.

Based on the genomic analysis of *Alicyclobacillus* sp. HJ, a putative 204 amino acid protein encoded by *orf0188* was predicted to be involved in detoxifying superoxide radicals, converting them to hydrogen peroxide and oxygen. Blast analysis indicated that *Aa*SOD exhibits 72.5% identity with a Mn-SOD in *Geobacillus stearothermophilus* (Adams et al., 2019) and 69.7% identity with the Mn-SOD in *Bacillus subtilis* 168 (Liu et al., 2007). As shown in Figure 1, *Aa*SOD was phylogenetically clustered with some other identified Mn-SODs and the Fe/Mn SODs from *Staphylococcus* species, while relatively distant with the Fe-SOD proteins. As both Mn- and Fe-SODs from *Pseudomonas aeruginosa* PAO1 or *E. coli* K12 were blasted as homologs of *Aa*SOD, the amino sequence of *Aa*SOD showed 56.8% identity with that of Mn-SOD (Steinman, 1978) from *E. coli* K12, and also 48.3% with its Fe-dependent SOD (Carlioz et al., 1988). This firmly verified the conclusion that Fe- and Mn-SODs are highly conserved in sequences and three dimension (3D), while possessing different dependence to metal ions (Lah et al., 1995). Based on the multiple sequence alignment, the Mn- and Fe/Mn SODs share more conserved sequences as shown in Figure 2, while possessing more variations from our Fe SODs. Consequently, we assumed that the catalytic process of *Aa*SOD may use Mn or Fe/Mn as its cofactors.

**FIGURE 3:**
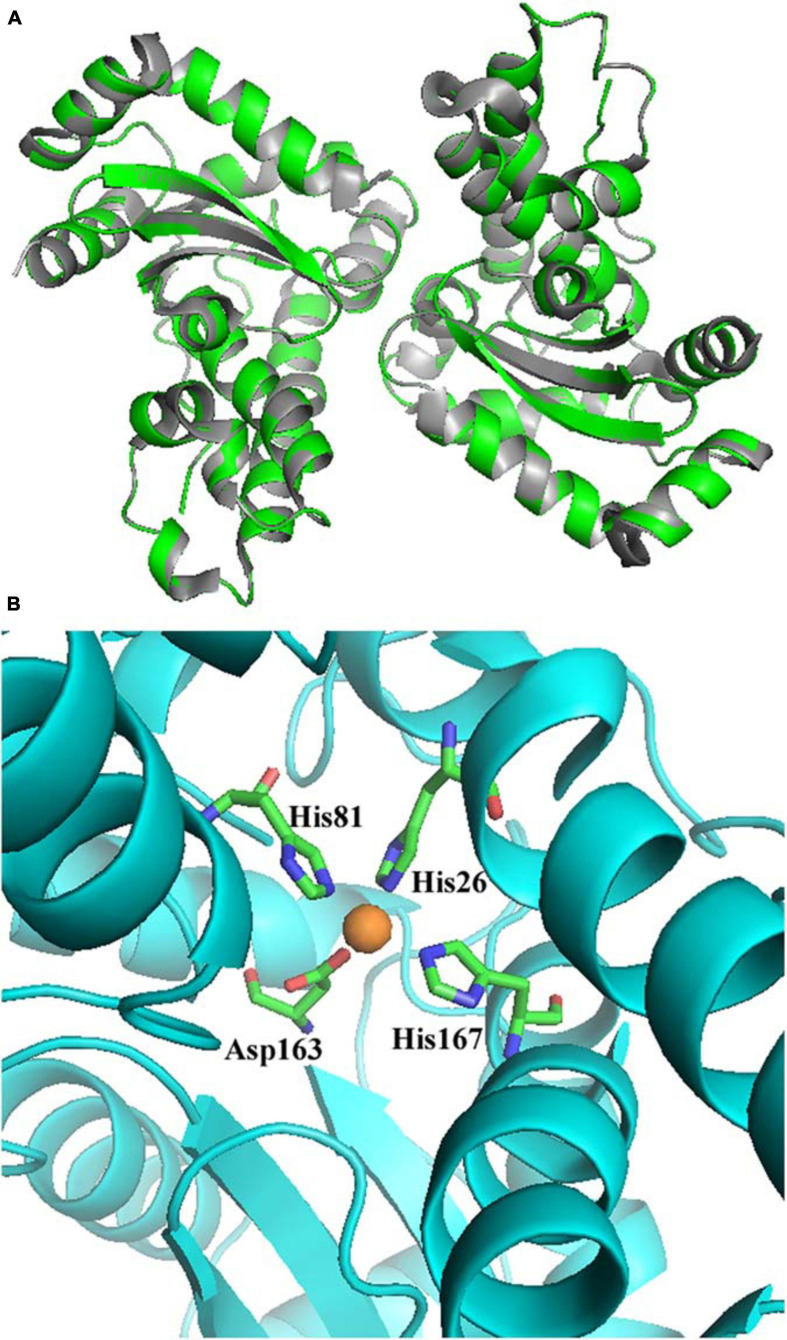
Superimposition of the predicted structure of *Aa*SOD (Green) over its template of *Geobacillus stearothermophilus Gs*-MnSOD (gray) **(A)** and four predicted residues for manganese binding **(B)**.

A homology model of *Aa*SOD was generated by the auto modeling system in the SWISS-MODEL server, based on the recently resolved structure of *Gs*-MnSOD (PDB: 6pro) as the template (Adams et al., 2019), which is from strain *G. stearothermophilus* and aligns 72.5% with the query sequence. As shown in Figure 3A, the superimposition of the predicted 3D structure of *Aa*SOD significantly conformed to the tertiary structure of *Gs*-MnSOD. The four residues for manganese binding, His 26, His 81, Asp 163, and His 167, are all well conserved in the *Aa*SOD sequence (Figure 2) and occupy identical spatial locations in its homologs structure (Figure 3B).

### Protein Purification

**FIGURE 4:**
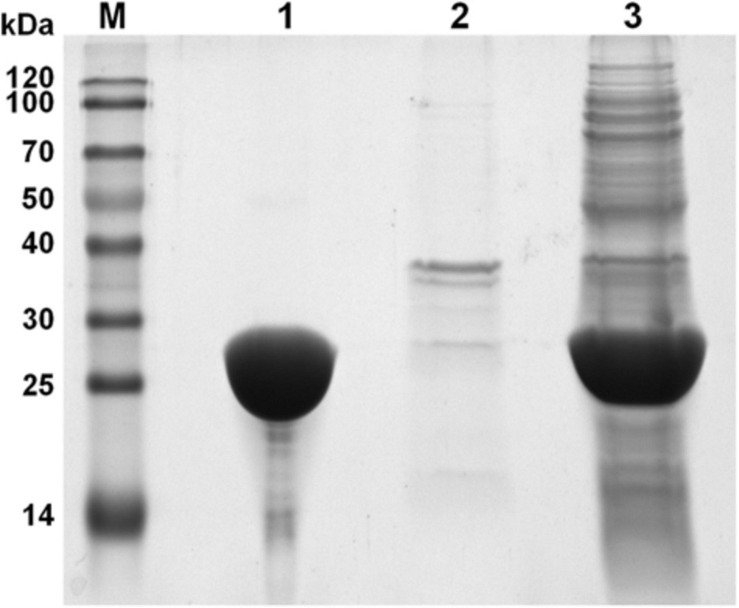
SDS-PAGE spectrum of *Aa*SOD. Lane M, Marker; lane 1, purified protein after His-Trap affinity column; lane 2, cell debris of the *E. coli* recombinant harboring *Aa*SOD; lane 3, supernatant of the *E. coli* recombinant harboring *Aa*SOD induced by IPTG for 12 h at 18°C.

To avoid the formation of inclusion body during protein expression process and gain high expression level, *Aa*SOD was heterologous expressed in the *E. coli* host of BL21 Codonplus, which contains extra copies of the argU, ileY, and leuW tRNA genes. In addition, the heterologous expression of *Aa*SOD was induced at a low temperature of 18°C. As a result, a large amount of SOD was produced after 10-h induction by shaking at 100 rpm. As shown in Figure 4, a tense band of approximately 25 kDa was detected in the SDS-PAGE, which is in accordance with the theoretical molecular weight of 22.82 kDa. After one-step purification on the His-Trap affinity column by 250 mM imidazole elution, a high yield of 99.8 mg l^–1^ protein with high purity of 95% were obtained. We assumed that the high purification level and recovery rate would be benefit from the high expression levels of the protein. This confers *Aa*SOD great potentials for scaled-up production and industrial applications. However, extracellular expression protocol could be needed for preparing *Aa*SOD products considering its purification efficiency. To choose host strains that are suitable for extracellular protein expression, such as *Bacillus subtilis*, would be addressed in the *Aa*SOD production.

### Metal Incorporation of *Aa*SOD

To explore the metal incorporation of *Aa*SOD in its native form, 1 mM MnSO_4_ and (or) FeSO_4_ was added into LB medium during the expression process. As shown in Table 1, the proteins purified from the LB medium without ion supplementation (native protein) possessed a specific activity of 90.94 U mg^–1^, which is obviously higher than the apo-enzyme (5.5 U mg^–1^, Table 1). The fact is that the natural LB medium contains trace metal ions, and thus provides Fe^2+^ or Mn^2+^ to the *Aa*SOD for cooperation. It was further confirmed by the presence of coordinated Fe^2+^ and Mn^2+^ in the ICP-MS analysis. In the natural *Aa*SOD, the Fe^2+^ and Mn^2+^ contents per one molar proteins were 0.051 and 6.85 × 10^–3^ mol, respectively. However, only 8.18 × 10^–4^ mol of Fe^2+^ and 5.20 × 10^–5^ mol of Mn^2+^ were detected in the apo-SOD. Different from the lower activity of proteins that expressed in the natural or Fe^2+^-rich LB medium, there was significantly higher *Aa*SOD activity of 1198.55 U mg^–1^ purified from the *E. coli* culture enriched with 1 mM MnSO_4_. And *Aa*SOD from the medium containing these two ions, displayed a modest activity of 534.56 U mg^–1^. Differently, the Fe^2+^-enriched cultures induced similar amount of *Aa*SOD but with very low activity (74.65 U mg^–1^), indicating that *Aa*SOD is a cambialistic enzyme because of Mn^2+^ binding being much more effective than Fe^2+^ binding, and therefore should be assigned into Fe/Mn type SOD. The activity bias between Fe- and Mn-bound SOD was also identified in some thermostable SODs, in which their Fe-reconstituted SOD activity is much lower than that of Mn-reconstituted SOD (Li et al., 2016). For examples, the SOD from thermophilic *C. aurantiacus* was most efficient with manganese incorporated, up to 30% of the activity was retained with iron (Lancaster et al., 2004). What’s more, the MnSOD_*cd*_ from *Clostridium difficile* exhibited the highest activity while Fe-sub-MnSOD_*cd*_ showed only 1/10 activity of MnSOD_*cd*_ (Li et al., 2014). In the ApeSOD of *A. pernix* K1, it was hypothesized that the Fe-bound mode could mimic the product-inhibited form by residue Tyr39, and was proposed as an explanation for the lower activity of Fe/SOD than Mn/SOD (Nakamura et al., 2011). Therefore, we suspected that the binding mode of metal cofactors in *Aa*SOD was similar as those SODs, in which Fe^2+^ competitively binds to the catalytic sites and thus restrains the activities of these cambialistic SODs. Therefore, it would be significantly beneficial to the SOD activity when we created an Mn^2+^-enriched environment for the expression of these Fe/Mn SODs, as our observations of high SOD activity in the Mn^2+^-enriched medium of this study.

**TABLE 1:** Superoxide dismutase (SOD) activity incorporating various ions.

Sample	Specific activity (U⋅mg^–1^)
Natural medium	90.94 ± 0.47
*Aa*SOD from Fe^2+^ enriched medium	74.65 ± 2.92
*Aa*SOD from Mn^2+^ enriched medium	1198.55 ± 20.36
*Aa*SOD from Fe^2+^ and Mn^2+^ enriched medium	534.56 ± 27.33
Apo-enzyme	5.50 ± 0.40
Fe^2+^-reconstitution	21.05 ± 1.10
Mn^2+^- reconstitution	517.63 ± 48.35
Fe^2+^/Mn^2+^- reconstitution	270.27 ± 127.83

*The apo-enzyme was prepared by dialyzing the purified AaSOD with 50 mM acetate buffer (pH 3.8) containing 8 M urea and 10 mM EDTA.*

Metal fidelity of *Aa*SOD was further investigated by an *in vitro* reconstitution experiment with 1 mM MnSO_4_ and(or) FeSO_4_. As a result, the metal specificities were highly consistent with corresponding proteins expressed in the MnSO_4_ or FeSO_4_ enriched medium. As shown in Table 1, the specific activities of all treatments displayed higher activities than the apo-enzyme. The *Aa*SOD sample that was reconstituted exclusively in the presence of Fe^2+^ had lower specific activity of 21.05 U mg^–1^, which was higher than the apo-enzyme while being lower than native *Aa*SOD (90.94 U mg^–1^) expressed in the crude LB medium. Similarly, the Mn^2+^-bound form of *Aa*SOD also exhibited a remarkably higher specific activity of 517.63 U mg^–1^, while the reconstituted sample upon both ions obtained a decreased activity of 270.27 U mg^–1^.

### Thermo- and pH-Stabilities of *Aa*SOD

**FIGURE 5:**
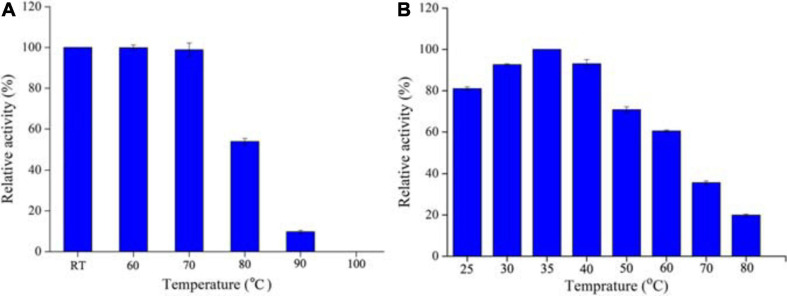
Thermostability **(A)** and optimal temperature **(B)** of *Aa*SOD. For thermostability measurement, the purified *Aa*SOD was incubated at different temperatures (RT, 60, 70, 80, 90, and 100°C) for 1 h, and subsequently measured the residual activities. The relative activity was calculated as the percentage of the maximum activity at 35°C. To achieve the optimal catalytic temperature, the activity of purified *Aa*SOD was tested at different temperatures (25–80°C) and the highest activity was set as 100% for relative activity calculation.

For industrial purpose, many enzymes are preferred to be processed into solid state, which could be realized by freeze drying or spray drying technology. In the case of freeze drying, the enzymes can preserve high activity under lower temperature. However, the large-scaled production is limited by its high energy consumption and processing cost. Thus, the spray drying process is more favorable and widely used. In addition, thermostable enzymes are often associated with robust resistance to harsh environments, including detergent, strong acid and alkali, or chemical and denaturants (Vieille and Zeikus, 2001). Therefore, thermostable SODs are of great interest and screened as good candidates for industrial production. As we previously described, to explore excellent SODs for industrial application, some thermostable SODs had been identified from thermophilic strains. *Alicyclobacillus* sp. HJ is a strain from hot-spring and possesses thermoacidophilic properties, i.e., grows optimally at pH 4.0 and 65°C. Therefore, it was regarded as an ideal source of thermostable or acid stable proteins, as our previous observation on a thermoacidophilic α-amylase (Zhang et al., 2018). Expectedly, *Aa*SOD exhibited high stability under high temperatures, with whole activity retaining after incubation at 60–70°C for 1 h (Figure 5A). In addition, 52.7% activity was still detected after incubation at 80°C for 1 h. Despite of robust thermostability, the optimal catalytic temperature of *Aa*SOD is mesophilic. During a range of temperatures of 20–70°C, the highest activity was observed at 35°C (Figure 5B). This is different from many SODs from thermopiles, which generally possess an optimum activity at high temperature (Seatovic et al., 2004; Zhu et al., 2013).

**FIGURE 6:**
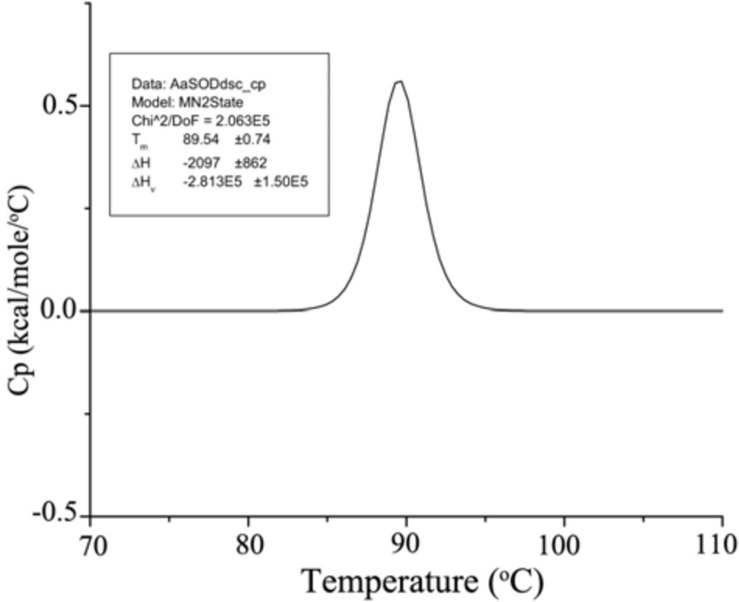
DSC spectrum of *Aa*SOD. The purified *Aa*SOD was scanned from a temperature range of 70–110°C at a rate of 1.5°C min^–1^. The transition curve was fitted by non-2-state model and the *T*_*m*_ value was calculated (MN2state) in the Origin software.

By directly measuring the forces stabilizing the conformational structure, DSC analysis is ideally suitable to evaluate protein thermal denaturation (Singh and Singh, 2004). Hence, the thermal denaturation of *Aa*SOD was further measured with DSC analysis, by scanning from 70 to 120°C based on the purified *Aa*SOD. After processes of buffer correction, normalization, and baseline subtraction, the DSC curve was fitted by the Gauss function (Figure 6). In the meanwhile, the melting temperature (*T*_*m*_) was calculated as 89.5°C and the Δ*H* value was −2097 cal mol^–1^. This denaturation temperature is somewhat higher than the incubation temperature, of which the maximal activity was observed (Figure 5B). To our best knowledge, the thermophilic SODs are generally active at high temperatures, e.g., SOD_ASAC from *Acidilobus saccharovorans* has an optimal activity at 70°C and denaturation temperature of 107.3°C (Slutskaya et al., 2012). Due to their vast bioavailability of SODs, they are widely used in cosmetics and food additives, as well as in pharmaceuticals (Bafana et al., 2011). Compared to the recorded thermostable or themophilic SODs, the thermostable but mesophilic properties of *Aa*SOD not only meet the requirement for the processing technology, but also endow its advantage to keep full functions after being taken into the organisms.

**FIGURE 7:**
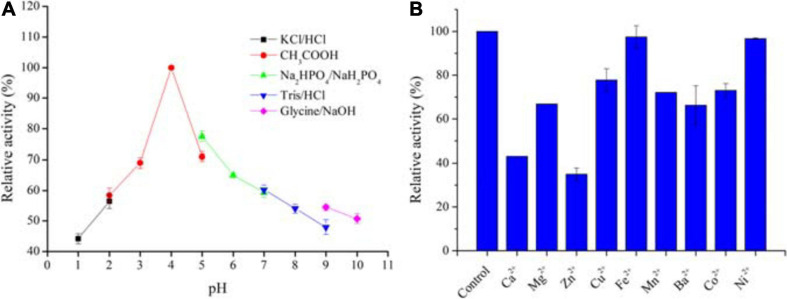
Stabilities of *Aa*SOD in buffers with different pH values **(A)** and effect of divalent ions on the *Aa*SOD activity **(B)**. The purified enzyme was incubated in the buffer systems of 200 mM KCl–HCl buffer (pH 1.0–2.0), acetate buffer (pH 2.0–5.0), sodium phosphate buffer (pH 5.0–7.0), Tris–HCl buffer (pH 7.0–9.0), and glycine–NaOH buffer (pH 9.0–10.0) for 1 h, 25°C. The maximal activity was taken as 100% for relative activity calculation. For metal ions effects, the purified enzyme was incubated with 1 mM of divalent metal ions in 50 mM Tris–HCl buffer (pH 8.0), at 25°C for 30 min. The mixture with no ions supplementation was used as a control for relative activity calculation.

To date, available methods for measuring SOD activity are almost based on inhibitive assay, which are seriously restricted to the reaction pH. Not unexpectedly, we failed to measure the acidic activities of *Aa*SOD, even at pH 6.0 conditions (data not shown). Therefore, we only tested the pH tolerance profiles by incubating the enzyme in various pH buffers for 1 h. Using this maximum activity as 100%, the relative activities of those proteins incubating in other pH buffers were calculated and shown in Figure 7A. Inspiringly, the enzyme showed good stability under a broad range of pH conditions with the highest stability at pH 4.0. In particular, it retained attractively high activities at extremely acidic conditions, with around 70 and 60% activities retained at pH 3.0 and 2.0, respectively. This provides compelling evidence that *Aa*SOD is an acidophilic enzyme, which is in accordance to our previously observations, that the α-amylase from strain *Alicyclobacillus* sp. HJ is also highly active at pH 4.0 (Zhang et al., 2018). To our best knowledge, no recorded SOD showing such extremely acidic tolerance so far, and thus *Aa*SOD would be served as an ideal model protein for exploring the mechanism of acidic tolerance. Moreover, abundant studies concluded that exogenous SOD supplementation has pharmacological and therapeutic benefits to mammals, including reduce liver oxidative stress in diabetic animals, resist inflammatory diseases, boost antioxidant defense and so on (Stephenie et al., 2020). Consequently, being an oral medicine or dietary supplement would be a favorable form for SOD administration. Regarding the advantage of excellent acidic tolerance, *Aa*SOD will be stable in the stomach after being digested by the organisms, and thus assure its activity as food additive and for medicine use.

### Influence of Divalent Ions on *Aa*SOD Activity

As shown in Figure 7B, various divalent metal ions were supplemented into the reaction mixture and tested for their influence on the catalytic activity of purified *Aa*SOD. As a result, the SOD activities were partly suppressed by some tested metal ions. However, still more than 60% activities were reserved upon tested metal ions, with exceptions of Zn^2+^ and Ca^2+^. As shown in Figure 7B, only around 40% activity was preserved in the presence of 1 mM of Zn^2+^. Differently, Fe^2+^ and Ni^2+^ showed no influence on the enzymatic activity, with the activity retained completely upon Fe^2+^ and 96.7% in the presence of 1 mM Ni^2+^. These results are mostly in agreement with the Mn-SOD from *Geobacillus* sp. EPT3, in which most metal ions except Mn^2+^ partially inhibited the enzyme activities but not lethal (Zhu et al., 2014). This guarantee high activities of these SODs when exposing to trace metal ions in applications.

## Conclusion

This manuscript firstly describes one thermostable and cambialistic SOD from a thermoacidophilic *Alicyclobacillus* strain. There are ample potentials in various applications with its advantages: it is significantly active in the mesophilic environment but highly stable under high temperatures, and it is an acidophilic protein with high stability at extremely acidic environments. Its large amount of heterologous expression and superior activity in the presence of Mn^2+^ create great feasibility for its industrial scale producing. Future studies would focus on optimizing its large-scaled expression and purifications protocols, achieving exogenous expression in the biosafety strain like *B. subtilis*, as well as experimentally testing its bioactivity and bioavailability in the animal models.

## Data Availability Statement

The nucleotide sequence encoding AaSOD was deposited in the GenBank database, accession number LC573735. The Strain Alicyclobacillus sp. HJ was deposited in the Marine Culture Collection of China, accession number MCCC 1K03506.

## Author Contributions

CY and XD conceptualized and defined the experimental design. XD, WW, HH, and SL performed the experiments. CY, HH, and PL carried out the data analysis and manuscript preparation. All authors contributed to the article and approved the submitted version.

## Conflict of Interest

The authors declare that the research was conducted in the absence of any commercial or financial relationships that could be construed as a potential conflict of interest.
